# A Work-Based, Fully Remote, and Peer-Supported Exercise Snack Behavior Change Intervention (MOV’D): Protocol for a Randomized Controlled Pilot Trial

**DOI:** 10.2196/64455

**Published:** 2025-08-07

**Authors:** Ashley Monteiro, Jessie Moore, Rocky Aikens, Angela Duckworth, James J Gross, Dan Schwartz, Mike Baiocchi, Judith J Prochaska, Marily Oppezzo

**Affiliations:** 1 Stanford Prevention Research Center Stanford University School of Medicine Palo Alto, CA United States; 2 Mathematica Princeton, NJ United States; 3 Behavior Change for Good Initiative University of Pennsylvania Philadelphia, PA United States; 4 Department of Psychology Stanford University Stanford, CA United States; 5 Graduate School of Education Stanford University Stanford, CA United States; 6 Department of Epidemiology and Population Health Stanford University Stanford, CA United States

**Keywords:** physical activity, sedentary behavior, eHealth, intervention, behavior change theory, exercise snacks, social support, exercise, behavior change, behavior, peer support, feasibility, acceptability, pilot study, Fitbit, self-monitor, sedentary

## Abstract

**Background:**

Prolonged sitting and lack of moderate to vigorous physical activity represent 2 independent risk factors for myriad poor health outcomes. The negative effects of prolonged sitting can be ameliorated with as little as 2 minutes of large muscle movement. Further, cardiovascular benefits from moderate to vigorous activity can be accumulated throughout the day in short bouts rather than require continuous long bouts. Taken together, “exercise snacks” provide a way to both interrupt prolonged sitting and accumulate moderate to vigorous physical activity during a sedentary workday.

**Objective:**

This protocol describes the feasibility and acceptability pilot of MOV'D (Move Often Every Day)—a fully remote, peer-supported behavioral intervention to interrupt prolonged bouts of sitting at work with exercise snack breaks.

**Methods:**

The MOV’D pilot study aims to recruit approximately 60-80 participants who work full-time in a sedentary occupation. Participants were randomly assigned either to the Fitbit Control or the MOV’D experimental group in a randomized 2-group design. The pilot study had a 4-week active intervention and a 4-week follow-up with assessments at baseline, 4 weeks, and 8 weeks. The Fitbit control group received a Fitbit to self-monitor their physical activity prior to receiving the intervention material at the end of the study. In addition to Fitbit, the MOV’D intervention features included a private social support group chat seeded with daily experimenter prompts, weekly 5-minute behavior change technique videos, daily self-monitoring, and daily exercise snack suggestion videos.

**Results:**

Study enrollment began in March 2022 and concluded in June 2022. Data collection concluded in October 2022. We enrolled 70 participants, and 68 participants completed all the study assessments.

**Conclusions:**

This protocol integrates findings from education, behavioral sciences, sedentary behavior, and exercise physiology to promote building an exercise snack habit at work: taking short intense exercise breaks to break up prolonged sitting. The results from this pilot study will show the feasibility and acceptability of the MOV’D intervention.

**Trial Registration:**

ClinicalTrials.gov CT05360485; https://clinicaltrials.gov/study/NCT05360485

## Introduction

### Background

Cardiovascular disease (CVD) is the leading cause of death in the United States [1,2]. One major risk factor for CVD is uninterrupted bouts of sitting, which can decrease lipoprotein lipase activity, heart rate variability, glucose management, and vascular function, and increase resting blood pressure and back pain [3-11]. Another CVD risk factor is inadequate levels of moderate to vigorous physical activity (MVPA), with 77% of the US adults failing to meet MVPA guidelines [12]. A growing body of evidence highlights how even short bouts of MVPA or “exercise snacks” can provide significant health benefits [13]. Conceptually, one can address both CVD risk factors at once by interrupting prolonged sitting with MVPA bouts to accrue MVPA minutes. To date, only a few interventions interrupt prolonged sitting at work with exercise snacks. Further, few studies have investigated how to utilize behavior change strategies to motivate these exercise snacks in the workplace, resulting in a lasting habit. We first cover a brief background on exercise snacks and then describe some behavior change techniques (BCTs) and social support features incorporated into this protocol.

### Exercise Snacks and Other Breaks in Sitting

Exercise snacks or brief bouts of MVPA (1-5 minutes) performed periodically throughout the day [14] can simultaneously interrupt prolonged sitting and increase accumulated MVPA minutes [15]. With 80% of the jobs in the United States being predominantly sedentary, exercise snacks can serve as an accessible way to increase workers’ levels of activity while still at work [16]. Although exercise snacks have only appeared in a few studies to date, adopting the behavior of exercise snacks into one’s daily routine has proven to be a more sustainable way to increase daily activity [17,18]. Exercise snacks have physical health benefits. One study showed similar significant improvements in maximal oxygen consumption (VO_2_ max) and cycling time trial after 6 weeks of either 3×20-second cycle sprints spread throughout the day to the same volume or 3×20-second bouts all within a single training session [16]. Compared to a time-matched long bout of MVPA, multiple shorter bouts exhibit similar cardiorespiratory fitness [19-21], glycemic control [7,22,23], exercise adherence [24], and weight loss [19,25].

Similarly, disruptions in prolonged sitting through stretching, walking, or standing can also confer psychological and cognitive benefits during a workday. A recent meta-analysis concluded that short breaks (cognitive, physical, and relaxation) of less than 10 minutes can improve participants’ well-being [26]. Specifically in worksite interventions, breaks involving movement were shown to successfully increase overall levels of physical activity, maintain productivity or work performance, and reduce muscle and joint pain and fatigue [27-29]. Sutherland et al’s [30] review of sedentary behavior interventions exemplified the need for high-quality research across different settings in this field. One worksite intervention showed a significant improvement in individuals’ exercise enjoyment, self-efficacy, and physical activity levels when people integrated exercise snacks at work [30]. Stork et al [29] compared 3 times/day single staircase sprints (sprint snacks) to a single bout of 3 staircase sprints (single session) within participants and found a more positive valence with a lower perceived effort in the sprint snacks, and 10 of the 14 participants preferred sprint snacks. Additionally, workers are more productive on days they engage in exercise at work compared to days when they do not [31].

Finally, exercise snacks are a behaviorally accessible way to increase MVPA minutes at work. Exercise snacks are more flexible compared to a singular longer bout of MVPA. With minimal time and space requirements, exercise snacks are not only easy to integrate into most workplaces and workdays but also adaptable and accessible, as the intensity, duration, and time occurrence of these bouts can be self-determined. Although existing workplace exercise snack interventions exhibit mostly positive outcomes, more research is needed to elucidate the best behavioral science support to encourage people to adopt exercise snacks at work as a long-term lifestyle habit.

### BCTs

Although we know exercise snacks can be beneficial, initiating and sustaining this habit is difficult [32]. BCTs are evidence-based strategies based in various behavior change theories. Michie et al [15] developed a taxonomy of 93 BCTs linked to sustainable behavior change in 2013 [29,33-50]. Studies that intentionally incorporate behavioral theories of change are more effective than those that do not [51]. This pilot study incorporated several BCTs such as self-monitoring, social support, social cognitive theory, and implementation intentions to enhance the effectiveness of outcomes by addressing and modifying individuals’ behaviors [29,33-50]. Further, in addition to building BCTs, we explicitly *teach* participants BCTs via short (4-5 minutes) educational, interactive videos, to add to their own behavior change toolkit.

### Social Support

Behavior change interventions that capitalize on existing social support or create a study-based social support system are more effective at changing and improving health behaviors [52]. Social media platforms can be an effective tool for encouraging social support in remote studies. Social media stands out for its capacity to disseminate health information widely and enables users to engage with others, contribute to content, and offer social support [13,20,22,23,53-55].

These positive effects of social support can also be seen in a previous study [45], Tweet2Quit, which was a Twitter-delivered intervention for smoking cessation. That study utilized a hybrid social media–based support group where participants were given a Twitter account and additionally paired with a buddy to both support and receive support for sustained abstinence from smoking [45,56-58]. As a result, Tweet2Quit exhibited 2 times more sustained abstinence for smoking cessation compared to other standard care control methods [45,56-58]. Further, Tweet4Wellness, a walking-break intervention based on Tweet2Quit, identified engagement in social support correlated with an increase in active hours and walking breaks [59]. Here, we are utilizing Tweet2Quit’s buddy pairing within a larger social support group to enhance learning and behavior change in increasing exercise snacks at work.

### MOV’D (Move Often Every Day) Intervention

This protocol for MOV’D (Move Often Every Day) is a fully remote, peer-supported, behavior change intervention that delivers BCT videos and exercise snack videos (2-5 minutes of moderate to vigorous activity) to a private social media support group of participants with the goal of interrupting prolonged sitting at work with MVPA. Common moves used in exercise snacks include running upstairs, squat variations, jumping movements, and other high-intensity exercises that require little to no equipment. In this pilot, the term “snacktivity” was utilized in participant-facing materials in place of exercise snacks. The study builds off the original pilot Tweet4Wellness [59], which tested the feasibility, acceptability, and preliminary efficacy of a novel Twitter-based walking break intervention with daily behavior change strategies and prompts for social support, combined with a Fitbit compared to a Fitbit-wearing only control group. The Tweet4Wellness study found that (1) social media engagement correlated with improvements in active hours and daily steps; however, engagement dropped off halfway through the study period; (2) poststudy interviews (Multimedia Appendix 1) and survey questions indicated the acceptability of the study but a desire for social support that was similar in socioeconomic status or baseline activity levels; (3) at follow-up, the improvement in the ratio of moving to sitting time was maintained. These findings led to modifications seen in the MOV’D design (eg, added a matched peer motivation coach; incorporated a learning-by-teaching element to improve uptake of the BCTs taught to participants; changing the walk breaks to exercise snacks to also tackle the problem of inadequate MVPA in those who due to time or resource constraints are unable to achieve adequate MVPA).

Here, we describe the protocol for the feasibility and acceptability pilot trial of MOV’D—a fully remote, peer-supported behavioral intervention to interrupt prolonged bouts of sitting at work with exercise snack breaks.

## Methods

### Overview

**Figure 1:**
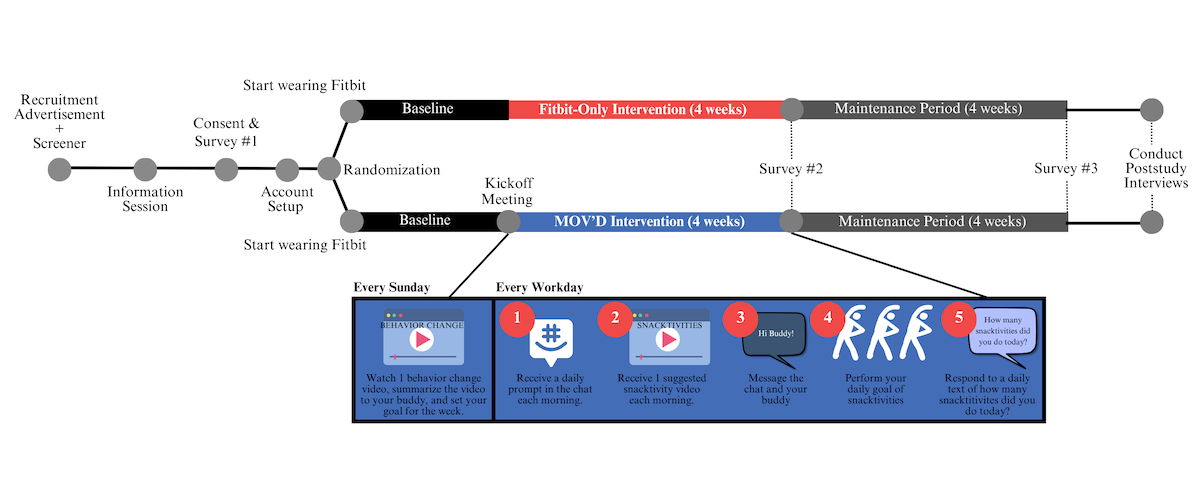
Study overview and timeline. MOV’D: Move Often Every Day.

This was a randomized controlled pilot testing feasibility and acceptability of the MOV’D intervention compared to Fitbit-only control. Data were collected in 3 waves or cohorts, allowing for an iterative design. The self-monitor only “Fitbit control” group established dropout rates as feasibility for a fully-powered trial. For the MOV’D intervention only, we will assess the average snacktivities reported for the 4-week intervention period. Both groups took part in a 4-week fully remote intervention with assessments at baseline, postintervention, and 4-week follow-up. An overview of the study design and timeline can be seen in Figure 1.

### Ethical Considerations

All procedures were registered at Clinicaltrials.gov (NCT05360485) and approved by Stanford University’s institutional review board (approval 60388) before beginning recruitment. All participants provided written electronic consent for study participation. Participants received US $10 e-gift cards for the completion of each follow-up survey (up to US $20) and their Fitbit device as study incentives. Data for this study were deidentified to ensure participants’ privacy.

### Participant Recruitment

Participants were recruited through a health care facility at a university; a staff email listserv; and a third party research service, ResearchMatch, a nonprofit program helping connect people interested in research studies with researchers [60]. Study advertisements included information about the study, critical inclusion criteria, and a link to our screener questionnaire. If interested, individuals would respond to our screener questionnaire to confirm eligibility.

### Inclusion and Exclusion Criteria

The inclusion criteria included being 25-75 years old, being sedentary for the majority of the workday, using social media and text messaging regularly, having a smartphone with unlimited texting, working full-time, being fluent in English, and demonstrating an interest in the study. Individuals were excluded if they had any medical conditions that would prevent them from increasing their physical activity, as determined by the Physical Activity Readiness Questionnaire [61]. Additionally, individuals were required to provide adequate contact information, could not have participated in Tweet4Wellness, and could not be currently enrolled in another physical activity or sedentary behavior study.

### Study Procedures

#### Information Session

After screening eligible, interested individuals were invited to a Zoom-based information session. This session overviewed the study procedures, timeline, and general research principles. Based on motivational interviewing principles, the session also covered randomization, the importance of hearing from participants especially if the study is not working for them, and the pros and cons of participating. This structure has been shown to improve participant trust, sense of partnership, and retention rates [62]. Participants could ask consent and study questions and could consent within 2 days of the information session. Participants were not blinded to the group assignments due to the nature of the study.

#### Buddy Pairs and Randomization

All participants were assigned a within-study buddy pair during randomization. We block randomized in 3 waves to ensure sufficient numbers for stratification. After 20 eligible participants consented, they were randomized to treatment by using statistical software [63], stratified by self-reported baseline physical activity levels using a block-4 design. Physical activity levels were measured using the International Physical Activity Questionnaire and calculated by summing up the metabolic equivalent of task minutes [64]. First, we stratified into groups of 4 by participants’ metabolic equivalent of task minutes. Second, we randomly assigned buddy pairs within each group of 4. Third, prior to randomizing pairs into treatment groups, adjustments were made to 4 buddy pairs to address strong mismatches in work environment or age while maintaining as close a match as possible on baseline physical activity levels. Next, the buddy pairs were randomized to treatment with equal probability by using statistical software. A group of 4 was used to account for the within-group buddy pairings. The second and third waves were randomized in a similar manner but accounting for uneven groups (groups not divisible by 4). Buddy pairs randomized to control were never informed of their buddy pair, while treatment buddy pairs were introduced via Zoom and encouraged to interact directly within the group chat. RA and MB generated the randomization scheme, while JM and MO enrolled the participants and allocated group assignments to the participants after randomization.

#### Account Setup Calls

Following randomization, participants had a Zoom visit with study staff for the study-provided account setup (both conditions: Fitbit accounts; MOV’D participants only: Twitter [cohort 1], GroupMe [cohorts 2-3]) and app downloads (both conditions: Fitbit; MOV’D participants only: Twitter [cohort 1], GroupMe [cohorts 2-3], EdPuzzle [all cohorts]). For MOV’D participants, Twitter or GroupMe was utilized for social support, and EdPuzzle was used to deliver the video materials (BCT videos and exercise snack suggestion videos). Due to updates at Twitter, our team switched social media platforms for cohorts 2-3. Additionally, study staff informed participants that they were to open their app daily to synchronize their data. Reminder messages were sent to participants on weekdays if they had not synchronized their data for 48 hours.

#### Intervention

Both MOV’D and Fitbit-only participants were instructed to wear their Fitbit device (Inspire 2) throughout the study, minimizing nonwear time. Participants in the Fitbit-only control groups were given Fitbit accounts with the reminders to move turned off. Participants in the MOV’D intervention group were given Fitbit accounts with the reminder to move turned on. Study staff monitored participants’ Fitbit accounts to ensure that reminders to move were not altered.

MOV’D participants received the following components (detailed in Table 1): (1) weekly BCT educational videos, (2) weekday daily reinforcement prompts sent in the group chat (Twitter or GroupMe) to reinforce the BCT content and increase within-group engagement, (3) weekday exercise snack video suggestion demonstrating 2-5 minutes of activity that the participants could do as their exercise snack break, (3) group support via access to a group of peers who are also in the study and attempting to incorporate exercise snack breaks into their daily work routine, and (4) nightly behavior tracking to collect data on the participants’ exercise snack breaks for that day and serve as an avenue of self-monitoring. Each week, MOV’D participants were sent their weekly BCT educational video content via a group chat message encouraging them to watch at least one of the provided videos each Sunday. There were 3-5 videos provided each week. MOV’D participants were asked to perform daily exercise snack breaks and actively participate in the group chat. On Sunday, participants set a daily goal of the number of exercise snack breaks for each workday for the upcoming week. Monday through Friday participants receive 2 messages each morning—1 message provided the encouraging daily reinforcement prompt and 1 message provided the suggested exercise snack video for that day. Participants were encouraged to engage in a group chat each day.

**Table 1:** Details of the MOV’D (Move Often Every Day) study components.

Study component	Delivery method	Delivery frequency	Description	Examples
Behavior change technique educational videos	Hyperlinked group chat message	Weekly (Sunday) in the evening	Short (4-5 minutes) videos that briefly describe a behavior change technique and how one can incorporate it into their daily life	Avoiding the “What the Hell” Effect: how to stop a slip up from becoming a dive bomb; Fix Faulty Thinking: how shifting your thoughts can help change your behavior
Daily reinforcement prompts	Group chat message	Daily (weekdays) in the morning	1-2 sentences that reinforce the ideas presented in the weekly behavior change technique videos and encourage engagement in the group chat	A situation plan for this week: add something to your workplace that makes it easier to do your snacktivities. What can you ADD to make snacktivities easier?
Exercise snack video suggestion	Hyperlinked group chat message	Daily (weekdays) in the morning	Short (2-5 minutes) follow-along exercise snacks video suggestions	4 min* Jump squat & Mountain climbers, Hip Hop Movement Break
Group support	Twitter (cohort 1); GroupMe (cohorts 2-3)	Daily (weekdays and weekends) throughout day	Cohort-size group (10-xx) chat, with buddy pairs within; encouraged to message every day	How did things go yesterday? It was a tough go for me, but I was still able to get 3 snacktivities
Exercise snack breaks (behavior)	Performed at work or at home	Participant-selected (1-5 times per day)	Weekly goal set on Sunday of how many exercise breaks they hoped to take each workday. They would utilize our videos or come up with their own exercise snacks	Walking briskly up and down the stairs for 3 minutes, YouTube video of the participant’s choosing, any of our exercise snack video suggestions
Behavior tracking	Text message	Daily (weekdays and weekends) in the evening	Daily autogenerated text messages prompt daily exercise snack tracking	How many snacktivities did you perform today?

### Measures

Assessments included 3 web-based surveys (conducted at baseline, 4 weeks [end of the intervention], and 8 weeks). Surveys assessed exercise self-efficacy, exercise mindset, self-reported physical activity, sleep, and other psychological variables across time points. Demographic information was collected at baseline. Study component acceptability was collected at 4 weeks.

### Feasibility and Acceptability

The measures for feasibility and acceptability are detailed in Table 2, including the predetermined cut point for success or target. Feasibility was tracked throughout the study with various methods of measurement. Acceptability metrics included qualitative acceptability data from poststudy interviews and responses to survey questions. Semistructured poststudy qualitative interviews were conducted with a subset of Fitbit-only control and MOV’D intervention participants.

**Table 2:** Definitions, methods, and targets of the feasibility and acceptability measures.

Outcome category, measure name		Method of measurement	Measure definition	Target
**Feasibility**				
	Recruitment rate	Recruitment data	The ratio of retained participants to interested individuals	>25%
	Recruitment of racial and ethnic minority individuals^a^	Recruitment data	The percentage of retained participants who were racial and ethnic minority individuals	>50%
	Retention rate	Surveys, Fitbit data	The ratio of retained participants to randomized participants	>80%
	Fitbit valid wear	Fitbit data	The percentage of participants with at least 80% of study days with valid Fitbit data	>80%
	Behavior change technique video adherence	Views of behavior change technique videos	The percentage of participants who watched at least 4 behavior change technique videos	100%
	Group support engagement	Chat logs from the group support group chat	The percentage of participants who chatted at least half (50%) of the study days	>80%
	Exercise snack video usage	Views of exercise snack videos	The percentage of participants who watched at least 4 exercise snack videos	100%
	Behavior tracking response rate	Message replies	The ratio of the replied messages to the sum of all the sent tracking messages	>90%
**Acceptability**				
	Qualitative acceptability	Semistructured interviews with participants	Interviews with participants at the end of the study asking about their experience with the intervention	N/A^b^
	Quantitative acceptability	Surveys	Survey questions asking about the acceptability of various study components on a 5-point Likert scale	>70% for all study components

^a^An individual who identified as African American, American Indian or Alaska Native, Chinese, Filipino, Indian, Japanese, Korean, Latinx or Hispanic, Pacific Islander or Native Hawaiian, Vietnamese, Other Asian, or other.

^b^N/A: not applicable.

### Exercise Snack Behavior Description

Exercise snacks are measured via self-report daily text messages for each workday. Participants were asked “How many snacktivities did you perform today?” Participants were instructed to reply with the number of completed exercise snacks or “NW” for any nonworking day.

### Other Measures

The Pittsburgh Sleep Quality Index [65] was utilized to assess sleep, the International Physical Activity Questionnaire [64] was adapted to measure physical activity levels, 2 questions were taken from the Rapid Assessment Disuse Index [66] to capture sedentary behavior, the Perceived Stress Scale [67] was used to assess stress levels, and the Need for Recovery Scale [68] was utilized as a proxy for workplace well-being. Additionally, self-reported height and weight were collected before and after the intervention. Demographic variables such as age, gender, racial and ethnic group, educational attainment, and questions about their work environment were collected at baseline.

### Analysis Plan

#### Feasibility and Acceptability Analysis

Qualitative acceptability interview data generated themes, discussed by the study team, and will be used to refine the intervention as needed. Interviews were transcribed and analyzed using an iterative approach based on reflective thematic analysis [69]. Consistent with the recommendations for behavioral pilot studies, we have set a priori standards for this pilot’s acceptability and feasibility metrics (see Table 2). Descriptive statistics for all feasibility and acceptability metrics were conducted using RStudio. As a pre-efficacy pilot trial (ORBIT model) [70], the primary objective is feasibility and acceptability. If successful, these data will inform the development of and provide estimates needed for the implementation of a phase III efficacy trial [70].

#### Exercise Snack Behavior Analysis

We will graph the average number of reported exercise snacks throughout the 4-week study and describe the average, standard deviation, and range of exercise snacks that are self-reported. As an exploratory measure, we will also examine the changes in the overall MVPA within participants.

## Results

**Figure 2:**
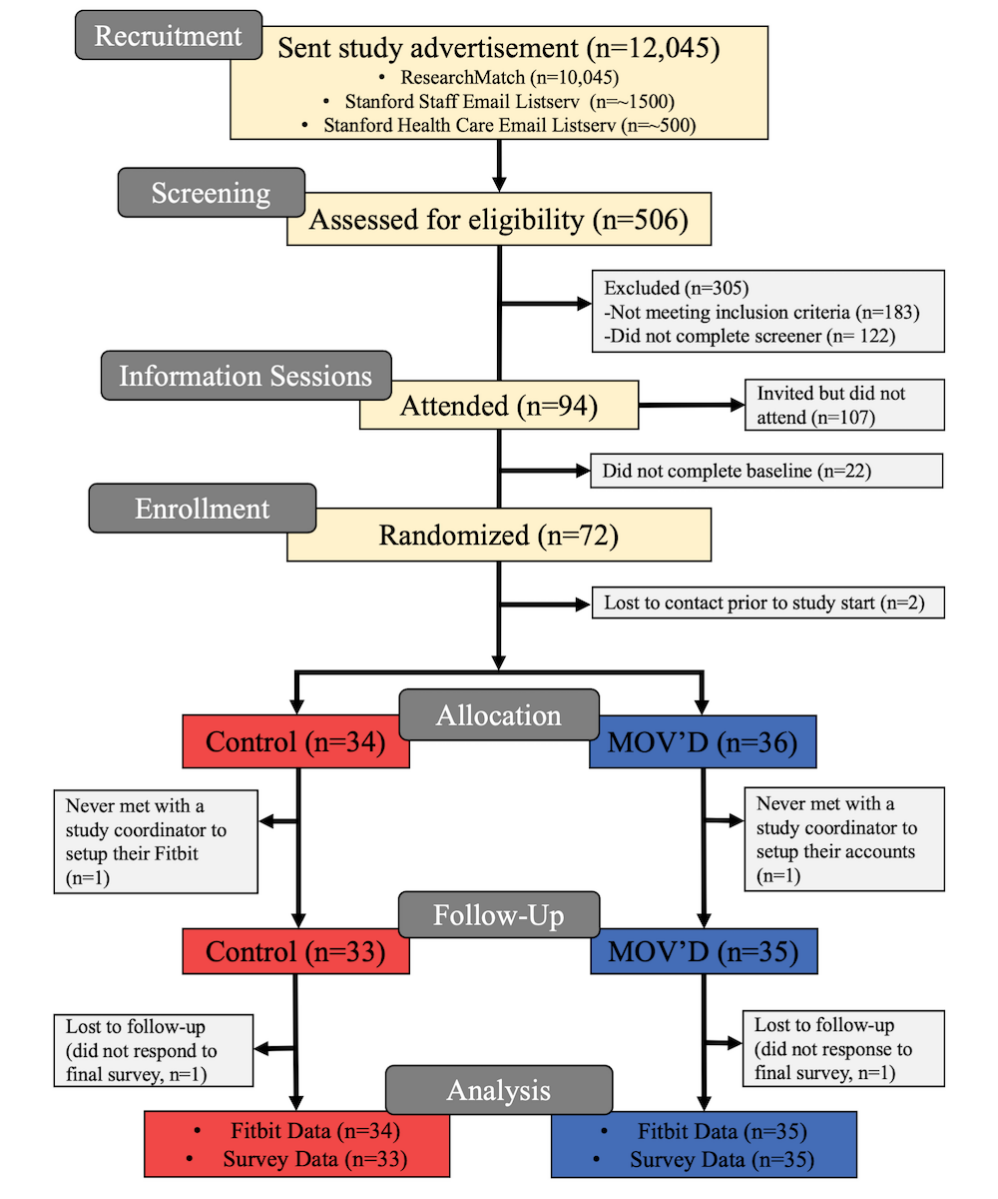
CONSORT (Consolidated Standards of Reporting Trials) flow diagram for the MOV’D study. MOV’D: Move Often Every Day.

Study enrollment began in March 2022 and concluded in June 2022. Data collection concluded in October 2022. As of July 2025, the data for this pilot study have been analyzed, and results are expected to be reported in late 2025. The MOV’D study has been updated, and an iterative version, MOV’D 2.0, began recruiting participants in 2024. Figure 2 shows the CONSORT (Consolidated Standards of Reporting Trials) diagram for this study (Multimedia Appendix 2).

## Discussion

This protocol paper describes a multicomponent, remotely delivered, and peer-supported behavioral intervention to increase exercise snacks at work. This intervention integrates findings from education, behavioral sciences, sedentary behavior, and exercise physiology to promote a habit of taking short intense exercise breaks to break up prolonged sitting. The study results will either inform an iteration to run another feasibility and acceptability pilot phase IIb study or inform a fully powered phase III efficacy trial.

The novel features of this intervention are augmented versions of previously established behavior change principles. First, exercise buddies have been shown in pilot trials to improve exercise adherence [71,72]. In MOV’D, buddy pairs are peer coaches. They not only provide accountability but teach each other behavior change strategies they learn each week. This increases the learning of each participant (eg, they watch 1 video but learn 2 strategies) and increases the likelihood of the coach remembering the strategy (eg, teaching another increases learning and application of concepts) [52,73]. Second, group support has been shown to increase the informational and emotional support for behavior change [74,75]. MOV’D augments this feature by integrating experimenter-seeded prompts to increase and guide engagement and reinforce behavior change practices. Third, exercise videos can provide guidance, aid in exercise completion, and provide role model behavior. MOV’D filmed diverse bodied and aged models in work attire and office settings to demonstrate the ability to do exercise snacks in atypical settings, modeling that a gym, equipment, or clothing are not requirements to integrate moderate to vigorous exercise bouts into one’s workday. This is based on the principles of the social learning theory [76] using role modeling and vicarious learning [77]. Finally, interventions based on the behavioral science theory are more effective than those that are not [51]. Typically, these evidence-based strategies are built into the intervention itself, such as reward incentives [78], study-provided reminders [79], or growth mindset modules [80]. The MOV’D intervention instead explicitly taught evidence-BCTs to participants to utilize themselves.

The ORBIT model of optimizing behavioral intervention development recommends iteration and pilot testing of feasibility and acceptability (ORBIT phase IIb, aim 2). This protocol and feasibility pilot will position the MOV’D intervention to either be further iterated or tested in an ORBIT phase III efficacy trial. The MOV’D intervention combines learning science, behavioral science practices, and social support in a fully remote, peer-supported workday activity intervention. The ultimate goal is to test a potential intervention addressing 2 independent and major risk factors for CVD: prolonged sitting and inadequate physical activity.

We expect this study to add to the literature on exercise snack workplace interventions. Stork et al [29] demonstrated preference for short exercise snacks. We add a measurement-only maintenance period after the intervention to investigate if the preference extends to behavior change without intervention scaffolds. We also add a nonintervention control group to test for differential dropouts.

A limitation of this study protocol is the inability to objectively measure heart rate and minutes of exercise snacks taken at work. Although pilot testing indicated the Fitbit may detect these, most consumer-grade wearables are inaccurate at higher heart rates with arm movements. Further, many of the exercise snack movements are done in place (therefore will not accrue steps on the wearable). Finally, Fitbit’s proprietary algorithms that smooth data may overwrite the short bursts of heart rates, detecting it as an anomaly. Another limitation is the potential between-group variability of group interaction and support. Groups can look different from each other in terms of the support offered, chattiness, and energy [81].

## Appendix

Multimedia Appendix 1 Poststudy interview guide.

Multimedia Appendix 2 CONSORT-eHEALTH checklist (V 1.6.1).

## Abbreviations

BCT: behavior change technique
CONSORT: Consolidated Standards of Reporting Trials
CVD: cardiovascular disease
MOV’D: Move Often Every Day
MVPA: moderate to vigorous physical activity

## Data Availability

The datasets generated and analyzed during this study will be available upon reasonable request from the corresponding author.
