# Supplementary material for: A Work-Based, Fully Remote, and Peer-Supported Exercise Snack Behavior Change Intervention (MOV’D): Protocol for a Randomized Controlled Pilot Trial
Source: JMIR Res Protoc. 2025 Aug 7;14:e64455. doi: 10.2196/64455 (PMC12371288; doi:10.2196/64455)
Supplement: Multimedia Appendix 1 [file resprot_v14i1e64455_app1.docx]

**Post-Study Interview Guide**

MOV’D Participant Interview Guide

1. General experience:

- Tell me about your experience with the MOV’D study.
- There were several features built in to help you change your behavior:
  - The planned videos;
  - the snacktivity suggestion videos;
  - the peer support group;
  - the break buddy support;
  - the nightly texts asking about your snacktivities

- Which feature(s) did you find most helpful?
  - How were they helpful?
- Which feature(s) did you find least helpful?
  - What would have made them more useful?
- Tell me about a time you were successful in achieving your snacktivity goal.
  - What helped you?
  - How did it feel doing the snacktivities in the middle of your work day?

1. Planned Videos:

- (if they watched the video) what was your favorite / most useful planned video strategy?
- What other strategies did you remember?
- (If appropriate) What would have made the videos more useful to you / easier for you to watch and learn from?

1. Snacktivity Videos

- (if they watched any snacktivity videos) what was your favorite?
- Did you find them useful?
- What would have made them more useful?

1. Group chat/buddy:

- How often did you participate in the group chat?
- What did you like about it?
- What did you wish would be different about it?
- Do you remember any of the daily prompts? (y/n)
  - If so, which ones?
  - If not, - ask whether they read the prompts at any point/ what would have been useful.
- How did you feel about having a break buddy?
  - (If appropriate) What would have made the experience stronger / better?

1. Nightly Texts:

- Did you find this useful?
- How did you track your snacktivities after the intervention ended?

1. Follow-up period:

- Tell me about your experience maintaining your habit in the month after the active peer support group and videos.
- Which plan strategies did you use?
- What were your biggest barriers?
- What could you have used during this period to make it easier to change your behavior?

1. Wrap up:

- Is there anything you particularly liked about the study / wished was different that we have not covered yet?
- What do you need / will you do going forward to help you sustain your snacktivity habit? (give behavioral coaching advice as needed)

Control Participant Interview Guide

- 1. General experience:
- Tell me about your experience with the MOV’D study.
- What was your experience when you found out you were in the Self-monitoring first group?
- Did you have a chance to read through the behavior change plan we emailed you?
- Do you have any questions about how to use the resources?
  1. Discussion of study features:

Talk through the various study features and how to utilize the resources that were emailed to them.

- The planned videos: these teach behavior change strategies; practice one a week
- The snacktivity suggestion videos: try to find one that you enjoy; these are just suggestions
- Self monitoring strategy: find a way to track your snacktivities that is sustainable and set weekly goals of how many snacktivities you aim to do per workday

1. Wrap up:

- Is there anything you particularly liked about the study / wished was different that we have not covered yet?
- What do you need / will you do going forward to help you start and sustain your snacktivity habit? (give behavioral coaching advice as needed)
